# The partial µ-opioid agonist buprenorphine in autism spectrum disorder: a case report

**DOI:** 10.1186/s13256-022-03384-w

**Published:** 2022-04-15

**Authors:** Charlotte Skoglund, Siri Leknes, Markus Heilig

**Affiliations:** 1 Division of Psychiatry, Department of Clinical Neuroscience, Karolinska Institute, Norra Stationsgatan 69, 113 64 Stockholm, Sweden; 2 Department of Psychology, University of Oslo Norway, Oslo, Norway; 3 Department of Diagnostic Physics, Oslo University Hospital, Oslo, Norway; 4 Center for Social and Affective Neuroscience (CSAN), Linköping University, Linköping, Sweden

**Keywords:** Autism spectrum disorder, Social cognition, Opioid, Buprenorphine, Attachment

## Abstract

**Background:**

There are currently no approved medications for impaired social cognition and function, core symptoms of autism spectrum disorder. We describe marked improvement of these symptoms with long-term low-dose administration of the partial µ-opioid agonist buprenorphine. We discuss these observations in the context of a role for endogenous opioid systems in social attachment, and theories integrating those findings mechanistically with autism spectrum disorder.

**Case presentation:**

M, a 43-year-old Caucasian male, is medically healthy. Despite social difficulties since childhood, he completed high school with better-than-average grades, but failed university education. A psychiatric evaluation in his twenties diagnosed attention deficit hyperactivity disorder but also noted symptoms of coexisting autism spectrum disorder. M accidentally came across buprenorphine in his late twenties and experienced progressively improved social functioning on a low daily dosage (0.5–1.0 mg/day), an effect maintained for 15 years. He lived independently and maintained a part-time occupation. After abrupt discontinuation of treatment, his autistic symptoms returned, and function deteriorated. Following evaluation by our team, buprenorphine was resumed, with gradual return to prior level of functioning. An attempt to formally evaluate M both on and off medication was agreed with him and approved by the Swedish Ethics Authority, but medication had to be resumed when the patient worsened following discontinuation.

**Conclusions:**

According to the µ-opioid receptor balance model, both excessive and deficient μ-receptor activity may negatively influence social behavior, and accordingly both opioid agonist and opioid antagonist treatment may be able to improve social functioning, depending on an individual’s opioid tone before treatment. Our case report is consistent with these hypotheses, and given the extensive unmet medical needs in individuals with autism spectrum disorders, randomized controlled trial appears warranted.

## Background

Autism spectrum disorder (ASD) is defined by functional impairments in social interaction, disruptions in social communication, and repetitive stereotyped behaviors [1, 2]. Patients typically experience an impaired ability to interpret and appropriately respond to social cues, as well as difficulties in initiating social contacts. This results in an impaired ability to cope with demands of daily life, and to reach long-term goals [3, 4]. ASD is associated with a plethora of coexisting psychiatric comorbidities, including attention deficit hyperactivity disorder (ADHD) [5, 6]. Although the degree of impairment varies considerably, ASD is generally associated with poor quality of life [7, 8], and increased morbidity and mortality [9]. With a worldwide prevalence of 1–2% [10–12], this represents a significant contribution to global disease burden.

There are currently no medications with regulatory approval or evidence for efficacy to treat any of the core symptoms of ASD. Two medications, risperidone and aripiprazole, are approved by the US Food and Drug Administration (FDA) for treatment of associated behavioral problems (e.g., aggression, severe temper tantrums) in ASD [13]. Although certain individuals may experience positive effects, these drugs are also associated with adverse psychological and physiological effects and their overall effectiveness has been questioned. In the absence of medications with an ability to target core symptoms of ASD, children and adults with this condition are frequently prescribed multiple psychiatric medications of questionable value [14–16]. Identifying medications targeting the core symptoms of ASD is therefore a major unmet medical need.

Here, we report a case of unexpected beneficial effects in ASD associated with long-term use of the partial µ-receptor agonist buprenorphine administered at a low dose. We then discuss this case in the context of the literature on the role of endogenous opioid systems for social cognition. With this as a basis, we outline opportunities for future research.

## Case presentation

M is a single, 43-year-old Caucasian male who lives partly with his aging parents and partly in an apartment provided by social services. He was first seen by author CS after his mother scheduled an appointment for him. The presenting complaint was that, after having achieved considerable functional improvement over more than a decade, the patient had experienced a functional decline in the months prior to the appointment. M and his mother, who accompanied him for the visit, jointly presented the history on multiple visits with author CS, an experienced consultant specializing in neuropsychiatric developmental disorders.

In addition to obtaining the history and carrying out a chart review, the patient was evaluated using an extensive battery of physical and neuropsychological assessments, based on international recommendations for diagnosing neurodevelopmental disorders. For the assessment battery, see Table 1. In addition to ASD-related assessments, this battery covers a broad range of psychiatric differential diagnoses, evaluates hazardous or harmful use of alcohol or drugs, assesses level of function in a broad range of domains, and assesses the patient’s perceived health-related quality of life.

**Table 1:** Assessments

Assessment	Area assessed	Refs.
The M.I.N.I. International Neuropsychiatric Interview (MINI)	A structured diagnostic interview for psychiatric diagnoses based on Diagnostic and Statistical Manual (DSM) criteria	[17]
Autism Diagnostic Interview—Revised	A semistructured diagnostic interview with parents and care-givers, detecting behaviors and symptoms associated with autism spectrum disorders in children	[18]
The Autism Diagnostic Observation Schedule, Second Edition (ADOS-2)	A semistructured, standardized observational assessment of communication, social interaction, and limited repetitive behaviors in diagnosing autism spectrum disorder in adults	[19]
The World Health Organization Disability Assessment Schedule (WHODAS 2.0)	A standardized method for measuring cross-border health and disability. It is based on a comprehensive set of questions in the International Classification of Functioning Conditions, Disabilities and Health (ICF) and is reliable and sensitive for measure of change after specific interventions	[20]
Alcohol Use Disorders Identification Test (AUDIT)	An extensively validated self-report questionnaire to screen for hazardous or harmful alcohol use; at appropriate cutoffs, it indicates a diagnosis of alcohol use disorder (AUD)	[21, 22]
Drug Use Disorders Identification Test (DUDIT)	A screening instrument for drug use, patterned on the AUDIT	[23]
The World Health Organization Adult ADHD Self-Report Scale V 1.1 (ASRS)	A self-report symptom checklist developed to have optimal concordance with the clinical classification of ADHD. The ASRS has shown to be a sensitive screener for identifying ADHD in adults seeking treatment for substance use disorders	[24]
Mood Disorder Questionnaire (MDQ)	A self‐report screening instrument with moderate to high sensitivity and specificity to identify bipolar disorder in psychiatric outpatients	[25]
The Patient Health Questionnaire (PHQ-9)	A self-report questionnaire to assess depression severity	[26]
EQ-5D (formerly EuroQoL) general health status	A self-report questionnaire to assess health-related quality of life	[27]

The patient was referred for a second opinion to author MH, a psychiatry professor and expert on addiction medicine, who reviewed medical records and the results of the initial evaluation. MH then met with the patient and his mother. Based on the totality of information, he determined that off-label prescription with low doses of buprenorphine could be justified, provided the patient with a prescription (2 mg once daily), and referred him back.

The patient encouraged the treatment team to share his experience with others through a scientific publication. To strengthen this reporting, it was agreed that a controlled discontinuation attempt would be carried out, allowing a formal evaluation of M both on and off buprenorphine. A protocol for this was developed and approved by the Swedish Ethics Authority (dnr. 2019-05335), and written informed consent was obtained. Based on the half-life of buprenorphine, M was instructed to discontinue his daily buprenorphine dose of 2 mg for 14 days, to allow for tests and assessments of psychosocial functioning off medication. As a rescue plan, the patient was to resume medication if he experienced unacceptable worsening.

### Demographic details and medical history

M is a single, 43-year-old Caucasian male residing in an apartment provided by social services. M was born with a patent foramen ovale (PFO), which was closed surgically in 2010. He has no cardiovascular symptoms and is otherwise medically healthy.

As a boy, M struggled with social interactions at school and experienced anxiety, inner restlessness, and subtle problems with sustained attention and concentration. With extensive daily support from his parents, he managed to complete middle and high school. While finding social situations difficult and exhausting, he reports that he appreciated the clear structure and objectives that school provided. He graduated high school with better-than-average grades.

In the words of his mother, after graduating high school, “M’s life stopped.” After several failed attempts at university studies, M’s parents sought psychiatric care. At the age of 22 years, he underwent a neuropsychiatric assessment and was diagnosed with ADHD. In addition, the chart states that M was diagnosed with “phobic and schizoid personality disorder.” A diagnosis of ASD was considered, but M’s social difficulties were thought to originate from “poor self-confidence due to motoric clumsiness and his adverse personality.” Because of the ADHD diagnosis, M was prescribed and tried several stimulant medications. He reports that he never experienced any subjective or objective beneficial effect from these. Instead, he exhibited adverse effects, such as further decreased cognitive flexibility, and obsessive–compulsive behaviors.

According to his own report, M has always believed that he had ASD in addition to ADHD. After several failed treatment attempts and progressive psychiatric and functional impairment, M started to experiment and self-medicated with various illicit drugs. He accidentally came across a batch of illicit buprenorphine and reports almost immediately noticing the positive effect of a low (0.8–2 mg) daily dose. At the time, his parents were unaware of his opioid use, but M’s mother reports that both parents did notice a significant improvement in M’s daily functioning and activity.

Specifically, M’s mother reports that he gradually showed more explicit and situation-congruent emotions and facial mimics, started making eye contact, was able to organize his room, created routines around food and exercise, and started seeing some long-lost high-school acquaintances. M reports that social interactions became more rewarding, and that, for the first time in his adult life, he was able to hold a meaningful occupation, first at a department store, and then at a dog shelter.

Having a strong sense of right and wrong, M felt uncomfortable hiding his use of illicit opioids from his parents. After he told them, they again sought medical attention, hoping to obtain a legitimate source of medication. After several rejections from public psychiatric care providers, they finally found a private practitioner who agreed to prescribe buprenorphine 1 mg/day off label.

M continued this treatment for about 10 years. He continued to show stable beneficial effects on social and executive functioning and was able to maintain his independent living and occupation. Over the course of this time, he did not escalate the dose or show any signs of tolerance or withdrawal. Buprenorphine treatment was abruptly discontinued when the prescribing physician had his prescription privileges for scheduled substances revoked.

At this point, M was unable to find a prescriber willing to continue off-label buprenorphine treatment. Meanwhile, according to both his own and his mother’s report, M’s level of functioning and quality of life once again declined rapidly. M expressed that “his life stopped without buprenorphine.” His aging parents were markedly distressed by the prospect that their son would not be able to function outside an institution once they were no longer alive. Before his first appointment, M had once more acquired a small amount of illicit buprenorphine and was rationing it at about 0.5 mg daily to make it last longer. He no longer had any employment or other meaningful occupation, had stopped his regular exercise, had moved back in with his parents, and was again depending on welfare.

### Diagnostic evaluation

On the basis of prior medical journals, screening forms, and longitudinal information from semistructured interviews with the participant, his parents, and relatives, the previously established ADHD diagnosis (F90.0C) was confirmed. In addition, the neuropsychiatric assessment could also establish a diagnosis of ASD (F84.0) with symptoms since childhood (before age 7 years) and in periods before starting on, as well as during periods off, buprenorphine.

### Resumed medication, and discontinuation trial

After he received his buprenorphine prescription, M submitted to regular urine screens. These excluded any concomitant use of illicit drug. He was compliant with the prescription, began to improve once more, and was able to return to his occupation at the dog shelter.

Upon discontinuing buprenorphine to allow assessment in a drug-free state, M did not report any clinically significant symptoms of opioid withdrawal. However, 1 week following discontinuation, he reported increasing social anxiety and impaired daily functioning. His symptoms become of a severity that the patient found unacceptable. He resumed buprenorphine medication at the prior dose before the scheduled neuropsychiatric reexamination.

## Discussion and conclusions

We describe here a patient with high-functioning ASD who experienced marked, stable, and independently confirmed long-term functional improvement with low daily doses (0.8–2.0 mg) of buprenorphine. Buprenorphine is a high-affinity partial agonist at µ-opioid and an antagonist at κ-opioid receptors. At the doses used here, it is estimated that buprenorphine has only a modest, perhaps 20%, central µ-opioid receptor occupancy (RO), and negligible activity at kappa receptors [28]. Thus, the clinical effects we report are likely to be associated with a low level of pharmacological µ-opioid receptor activation.

Endogenous opioid systems, and µ-opioid receptors in particular, have long been hypothesized to play a role in social processes and attachment [29]. Data indicate that a role for µ-opioid signaling in social function is phylogenetically conserved. For instance, µ-opioid receptor activation underpins the rewarding properties of social play in juvenile rats [30], and µ-opioid signaling is necessary for pair-bonding in the socially monogamous prairie voles [31]. In nonhuman primates, functional genetic variation in the gene encoding the µ-receptor moderates distress-related behaviors upon maternal separation of infants [32]. In healthy humans, a single dose of an opioid drug sharpened social preferences [33], increased visual attention to eyes [34], memory for happy faces [35], and ratings of positive social stimuli [36]. For negative social stimuli, acute opioid drug effects dampen attention to [36, 37] and perception of [38] negative facial expressions. Conversely, acute opioid antagonism is reported to reduce behavioral and brain responses to socially rewarding stimuli [34, 39]. However, it is also equally true that opiates in high doses can blunt sociality, resulting in introvert behaviors and anhedonia [40].

Research on the role of opioids for social attachment in particular was pioneered by Jaak Panksepp [41]. By extension from these data, Panksepp then proposed that autism could be caused by excessive brain opioid activity [42]. This has, however, not been supported by data; antagonist treatment has shown modest or no effects on core symptoms in clinical trials of children on the autism spectrum [43]. In fact, mice genetically modified to lack the µ-opioid receptor (rather than having an excessive activation of it) show severe social deficits [44] as well as stereotyped and perseverative behaviors necessary to fulfill ASD criteria [45]. These knockout mice also show a number of comorbid symptoms of ASD such as aggression, anxiety, and motor clumsiness, and are currently used as a mouse model of autism [46].

**Fig. 1:**
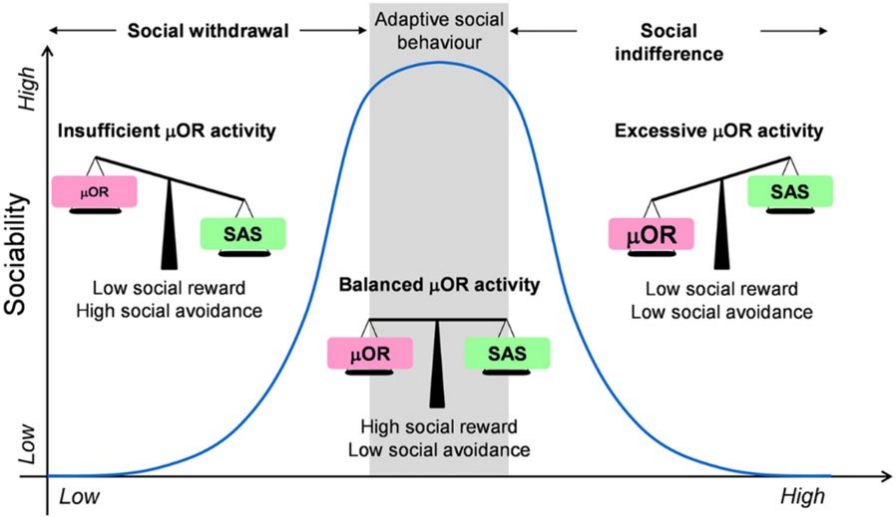
A schematic of the µ-opioid receptor balance model

An attempt to reconcile these seemingly contradictory observations is offered by the µ-opioid receptor balance model [46], which posits that both excessive and deficient μ-receptor activity may negatively influence social behavior through an inverted-U relationship (Fig. 1). Accordingly, the model predicts that both opioid agonist and opioid antagonist treatment may be able to improve social functioning, depending on an individual’s opioid tone before treatment.

Our observations in the case of M are consistent with this possibility. Since beginning to work on this case, we have been informally approached by colleagues who have made similar observations. Importantly, however, buprenorphine and other opioids are controlled substances that may be abused, diverted, and result in tolerance and addiction [47]. Given the current severe and lethal opiate epidemic [48], prescription of scheduled substances such as opioid should always be made by experienced clinicians and after careful assessment of individual potential risks and benefits.

Clearly, our noncontrolled clinical observations do not amount to evidence of efficacy. ASD is a heterogeneous condition, highly prevalent with other psychiatric and neurological disorders that may present with symptoms ranging from discrete social challenges to severe and global functional impairments [2]. Clearly, a case observation as described above does not allow for generalizability or treatment recommendations, in neither adult nor pediatric populations. However, our results do allow us to generate the hypothesis that low-dose buprenorphine may be a future therapeutic option in specific cases of ASD. Given the extensive unmet medical needs in this condition, putting this hypothesis to test through a randomized controlled trial appears warranted.

## Data Availability

Data sharing is not applicable to this article as no datasets were generated or analyzed during the current study.

## Abbreviations

ASD: Autism spectrum disorder
ADHD: Attention deficit hyperactivity disorder
